# Cerebral Perfusion Unveiled: A Comprehensive Review of Blood Pressure Management in Neurosurgical and Endovascular Aneurysm Interventions

**DOI:** 10.7759/cureus.53635

**Published:** 2024-02-05

**Authors:** Shubham Petkar, Vivek Chakole, Roshan Nisal, Vishnu Priya

**Affiliations:** 1 Anaesthesiology, Jawaharlal Nehru Medical College, Datta Meghe Institute of Higher Education and Research, Wardha, IND; 2 Anaesthesiology, awaharlal Nehru Medical College, Datta Meghe Institute of Higher Education and Research, Wardha, IND

**Keywords:** autoregulation, hemodynamic control, endovascular aneurysm interventions, neurosurgical interventions, blood pressure management, cerebral perfusion

## Abstract

This comprehensive review delves into the intricate dynamics of cerebral perfusion and blood pressure management within the context of neurosurgical and endovascular aneurysm interventions. The review highlights the critical role of maintaining a delicate hemodynamic balance, given the brain's susceptibility to fluctuations in blood pressure. Emphasizing the regulatory mechanisms of cerebral perfusion, particularly autoregulation, the study advocates for a nuanced and personalized approach to blood pressure control. Key findings underscore the significance of adhering to tailored blood pressure targets to mitigate the risks of ischemic and hemorrhagic complications in both neurosurgical and endovascular procedures. The implications for clinical practice are profound, calling for heightened awareness and precision in hemodynamic management. The review concludes with recommendations for future research, urging exploration into optimal blood pressure targets, advancements in monitoring technologies, investigations into long-term outcomes, and the development of personalized approaches. By consolidating current knowledge and charting a path for future investigations, this review aims to contribute to the continual enhancement of patient outcomes in the dynamic field of neurovascular interventions.

## Introduction and background

Cerebral perfusion, the delivery of blood to the brain, is a critical physiological process vital for the sustenance of neural tissues. It involves the intricate regulation of blood flow to meet the metabolic demands of the brain cells, ensuring an optimal supply of oxygen and nutrients. The brain's unique sensitivity to alterations in blood flow underscores the importance of maintaining a delicate balance to prevent ischemic or hypoxic damage [1]. The regulatory mechanisms governing cerebral perfusion are multifaceted, with autoregulation playing a pivotal role. Autoregulation allows the brain to maintain a relatively constant blood flow across various systemic blood pressures. Understanding these mechanisms is essential for tailoring interventions that optimize cerebral perfusion while mitigating the risks associated with neurosurgical and endovascular procedures [2].

The significance of precise blood pressure management becomes particularly pronounced in the intricate landscape of neurosurgical and endovascular aneurysm interventions. The brain's vulnerability to changes in blood pressure, both hypo- and hypertension, necessitates meticulous control during these procedures [3]. Neurosurgical interventions, such as clipping and bypass procedures, inherently disturb the natural hemodynamic equilibrium. Similarly, endovascular approaches, including coil embolization and flow diversion, introduce unique challenges to cerebral perfusion dynamics. Failure to manage blood pressure effectively during these interventions may lead to adverse outcomes, ranging from ischemic complications to hemorrhagic events [4].

This review endeavors to provide a comprehensive analysis of blood pressure management in the context of neurosurgical and endovascular aneurysm interventions. By delving into the nuances of cerebral perfusion and its implications for these procedures, the aim is to synthesize existing knowledge, identify gaps in current understanding, and propose avenues for future research. Through a systematic exploration of the available literature, this review seeks to contribute to refining clinical practices, ultimately enhancing patient outcomes in the intricate field of neurovascular interventions.

## Review

Neurosurgical aneurysm interventions

Overview of Neurosurgical Approaches

Clipping procedures: Aneurysm clipping treats brain aneurysms, balloon-like bulges of an artery wall. The procedure involves placing a small metal clip across the neck of the aneurysm to seal it off from the blood flow, preventing it from bursting or spilling blood into the brain [5,6]. The surgeon makes a small opening in the skull to access the brain and then uses an operating microscope and tiny instruments to perform the detailed surgical procedure [7]. Clipping of brain aneurysms has been available longer than endovascular therapy and has excellent long-term results [6]. The procedure is performed on both ruptured and unruptured aneurysms, but in the case of a ruptured aneurysm, it is performed on an emergency basis [8]. The choice of treatment depends on various factors, including the specific condition being treated, the patient's overall health, and the expertise of the medical team [9,10].

Bypass procedures: Intracranial aneurysms (IAs) can be treated using surgical treatments such as microsurgical clipping with or without bypass techniques and endovascular methods such as coiling, balloon- or stent-assisted coiling, intravascular flow diversion, and intravascular flow disruption [11-13]. Surgical clipping involves placing a small metal clip across the neck of the aneurysm to seal it off from the blood flow, preventing it from bursting or spilling blood into the brain [11-13]. Bypass techniques are also used in some cases, which involve rerouting blood flow around the aneurysm using a graft or vessel from another body part [11]. Endovascular techniques are less invasive and involve inserting a small catheter into an artery and guiding it up into the arteries of the neck using X-rays. Detachable coils are threaded through the catheter and placed in the aneurysm to fill it, effectively reducing or cutting blood flow into it [13,14]. Treatment choice depends on various factors, including the specific condition being treated, the patient's overall health, and the medical team's expertise [11-13].

Hemodynamic Considerations in Neurosurgery

Autoregulation and its role: The role of autoregulation in neurosurgery is crucial for maintaining stable cerebral blood flow and preventing hemodynamic disturbances. Hemodynamic neurosurgery considerations encompass managing blood pressure, intracranial pressure (ICP), and cerebral blood flow to ensure optimal conditions for the brain during surgical procedures. Several studies and articles discuss the impact of hemodynamic changes on neurosurgical patients, emphasizing the importance of maintaining appropriate blood pressure and ICP levels to support cerebral perfusion. One study published in the *Journal of Anesthesia & Analgesia* summarizes intraoperative blood pressure changes in neurosurgical patients, highlighting the significance of monitoring and managing blood pressure during these procedures [15]. Additionally, a narrative review published in the *Annals of Rehabilitation Medicine* focuses on hemodynamic considerations in the context of intraoperative neurophysiological monitoring during neuromuscular scoliosis surgery, emphasizing the relationship between hemodynamic parameters and neurophysiological changes [16]. Furthermore, the impact of position-dependent hemodynamic changes in neurosurgical patients is discussed in a review article, emphasizing the influence of patient positioning on intracerebral blood volume and ICP [17]. These sources underscore the importance of comprehensive hemodynamic management in neurosurgery, encompassing factors such as blood pressure, ICP, and cerebral blood flow to support optimal surgical outcomes and minimize the risk of hemodynamic disturbances.

Cerebral perfusion pressure (CPP): CPP is critical in managing patients with intracranial pathology, including traumatic brain injury and hemodynamic distress such as shock. CPP is the net pressure gradient that drives oxygen delivery to the brain, and normal CPP lies between 60 and 80 mm Hg [18,19]. Appropriate CPP is crucial in neurosurgery to ensure stable cerebral blood flow and prevent hemodynamic disturbances. Hemodynamic neurosurgery considerations encompass managing blood pressure, ICP, and cerebral blood flow to ensure optimal conditions for the brain during surgical procedures [17,20]. Several studies and articles discuss the impact of hemodynamic changes on neurosurgical patients, emphasizing the importance of monitoring and managing blood pressure and ICP levels to support cerebral perfusion [18,19,21]. CPP management techniques, such as systemic vasopressors, cerebrospinal fluid drainage, and mannitol administration, have maintained CPP levels in neurosurgical patients [21]. The choice of CPP management technique depends on various factors, including the specific condition being treated, the patient's overall health, and the medical team's expertise [21].

Blood Pressure Management Strategies

Preoperative considerations: Blood pressure management is crucial in neurosurgical aneurysm interventions, particularly in treating patients with aneurysmal subarachnoid hemorrhage (aSAH). Studies have shown that blood pressure management is essential in preventing hemodynamic disturbances and improving patient outcomes [22]. Preoperative considerations for blood pressure management may include using antihypertensive medication to lower blood pressure levels without compromising cerebral perfusion [22]. Blood pressure should be monitored continuously via an arterial line and documented regularly during the procedure [22]. Additionally, a risk assessment should be carried out to determine whether surgery is necessary, and preventative surgery is usually only recommended if there is a high risk of rupture [23]. Treatment choice depends on various factors, including the specific condition being treated, the patient's overall health, and the medical team's expertise [23-25].

Intraoperative blood pressure targets: In neurosurgical aneurysm interventions, intraoperative blood pressure management is crucial, particularly in treating patients with aSAH. Studies have emphasized the significance of maintaining appropriate blood pressure levels to prevent hemodynamic disturbances and improve patient outcomes. Blood pressure should be monitored continuously via an arterial line and documented regularly during the procedure. In the context of aSAH, there is a possible association between blood pressure levels and the risk of delayed cerebral ischemia, highlighting the importance of precise blood pressure management during these interventions [24,25]. Additionally, observations have indicated that high blood pressure after aSAH is related to poor outcomes, and treating high blood pressure can reduce the risk of rebleeding [24]. Therefore, maintaining optimal intraoperative blood pressure targets is essential for ensuring favorable outcomes in neurosurgical aneurysm interventions, particularly in aSAH.

Postoperative management: Postoperative blood pressure management in patients who have undergone neurosurgical aneurysm interventions, particularly those with aSAH, is a critical aspect of care. International guidelines recommend specific blood pressure targets for postoperative management, particularly in aSAH. For instance, guidelines suggest inducing hypertension in individuals with delayed cerebral ischemia while refraining from making such a recommendation due to a lack of evidence in other cases [25]. Observations have indicated a possible association between increased blood pressure and the risk of delayed cerebral ischemia, emphasizing the need for precise postoperative blood pressure management in these patients [22]. Additionally, antihypertensive treatment may decrease the risk of rebleeding by reducing the pressure against the weakened wall of the aneurysm [25]. Therefore, meticulous postoperative blood pressure management is essential in optimizing patient outcomes and minimizing the risk of complications following neurosurgical aneurysm interventions, particularly in the context of aSAH.

Endovascular aneurysm interventions

Overview of Endovascular Approaches

Coil embolization: Endovascular coil embolization is an option for treating ruptured and unruptured IAs. It involves placing a coil inside the aneurysm to prevent blood from filling it, ultimately blocking the aneurysm. This method is less invasive than surgical clipping and has shown promising results, with a shorter recovery time than traditional surgery. However, there is a higher risk of aneurysm recurrence with endovascular approaches, and routine angiograms may be recommended to identify any early regrowth of aneurysms [26,27]. Advancements in endovascular technologies and techniques have enabled the treatment of various cerebral aneurysms. Specific endovascular procedures include coiling, stenting, liquid embolization, and flow diversion. These procedures are performed from inside the patient's blood vessels and continue to evolve with the development of new technologies [14,28].

Flow diversion: Endovascular treatment is a less invasive procedure than surgical clipping for cerebral aneurysms, and it involves accessing the aneurysm by threading a small catheter through the arteries until the tip of the catheter reaches the aneurysm. Specific endovascular procedures include coiling, stenting, liquid embolization, and flow diversion. Flow diversion is a technique that involves placing a stent-like device across the neck of the aneurysm to divert blood flow away from the aneurysm sac, promoting thrombosis and eventual occlusion of the aneurysm. The first flow diverter introduced into the neuroendovascular space was the Pipeline Embolization Device (PED). Since then, many flow diverters have entered the neuro-interventional field, including the Silk, Surpass, and Flow-Redirection Endoluminal Device (FRED) [14,23,29,30].

Hemodynamic Considerations in Endovascular Procedures

Impact on cerebral perfusion: Endovascular procedures for cerebral aneurysms can impact cerebral perfusion. In a study, cerebral perfusion was measured before and after endovascular or surgical treatment of ruptured cerebral aneurysms, and disturbances in cerebral perfusion were common before and after treatment [31]. Another study evaluated hemodynamic changes after successful endovascular recanalization in patients with chronic intracranial artery occlusion and found that cerebral blood flow increased significantly in the perforating artery territory and cortical artery [32]. Cerebral hemodynamic evaluation after recanalization therapy can help identify patients with high risks of reperfusion-associated complications, and either hypo- or hyperperfusion may result in poor clinical outcomes [33]. In an exploratory study, high systolic blood pressure and mean arterial pressure during the pre-recanalization phase were found to have detrimental associations with functional outcomes after endovascular stroke treatment [34].

Challenges specific to endovascular interventions: The challenges specific to endovascular interventions in terms of hemodynamic considerations include maintaining hemodynamic stability and preserving perfusion to vital organs, particularly the brain, during the procedure. Patients undergoing endovascular procedures, such as those for abdominal aortic aneurysm repair, may present challenges to anesthesiologists due to their age, comorbidities, and the need for careful hemodynamic management [35]. Additionally, in the context of endovascular stroke treatment, the management of blood pressure during the procedure is crucial, as both excessively high and low systemic blood pressure levels can have implications for cerebral perfusion and patient outcome [34]. Furthermore, endovascular techniques, such as flow diversion, can impact cerebral hemodynamics, and evaluating cerebral perfusion before and after such interventions is an essential aspect of patient care [32].

Blood Pressure Management Strategies

Pre-procedural optimization is critical in ensuring patient safety and minimizing risks associated with anesthesia and surgery, particularly in endovascular procedures. This involves carefully balancing the risks of anesthesia, the treatment itself, and the potential consequences of delaying the procedure for individual patients [36]. In the realm of perioperative blood pressure management, personalized goals are crucial for preventing complications during surgery, including stroke, myocardial infarction, and renal failure. These goals are tailored to each patient's unique physiological demands and risks associated with the perioperative period, ensuring an individualized approach that maximizes patient safety [36].

The preoperative evaluation process relies on comprehensive assessments, utilizing ambulatory and home blood pressure monitoring to diagnose hypertension. Ambulatory blood pressure monitoring is recommended for patients with measurements exceeding 140/90 mmHg in community settings, offering a more accurate assessment by providing an average value over the monitoring period. This precision aids in identifying patients who may benefit from targeted preoperative interventions to optimize blood pressure levels before surgery [37]. In cases where preoperative blood pressure exceeds 180/110 mmHg, a cautious approach is recommended, recommending elective surgery's ideal delay. This delay allows for the initiation of antihypertensive treatment, aiming to bring blood pressure within safer ranges before the surgical intervention. However, ongoing debates surround the necessity of such delays, with some studies suggesting potential unnecessary postponements of elective surgeries based on older classifications of severe hypertension [37].

Effective communication between healthcare providers is emphasized, particularly when hypertension is newly identified during preoperative assessment. Timely relay of this information to the patient's primary care practitioner ensures continuity of care. It enables the primary care team to address and manage hypertension beyond the perioperative period, fostering a collaborative and patient-centered approach to healthcare [37,38]. Intraoperative blood pressure management focuses on maintaining blood pressure within a specific range throughout the surgical procedure. Anesthesiologists are pivotal in adjusting intraoperative blood pressure goals based on individual patient risk factors and medical history, contributing to more precise and tailored interventions. This personalized approach recognizes the unique characteristics of each patient and optimizes blood pressure management in the perioperative period [39].

Intraoperative considerations involve utilizing preoperative blood pressure to guide blood pressure management during the procedure and in the post-anesthesia care unit (PACU). Guidelines, such as those from the UK National Institute for Health and Care Excellence (NICE) and the American College of Cardiology/American Heart Association (AoA/BHS), provide recommendations for managing preoperative hypertension and its implications for intraoperative blood pressure management. Machine learning algorithms and individualized blood pressure targets based on baseline characteristics represent recent advances in predicting and managing intraoperative blood pressure changes [37-39,40]. Post-procedural care for blood pressure management is crucial, involving monitoring the patient's blood pressure to prevent complications. Careful attention is given to maintaining appropriate systemic blood pressure levels in the context of endovascular procedures, as extremes can impact cerebral perfusion and patient outcomes. The American Heart Association guidelines recommend specific systolic blood pressure targets based on the procedure's success, emphasizing the importance of careful monitoring and treatment adjustment for safe and effective perioperative hypertension management [41].

Comparative analysis

Neurosurgical vs. Endovascular Approaches

Efficacy in aneurysm treatment: The efficacy of endovascular treatment compared to neurosurgical approaches for cerebral aneurysms is a topic of ongoing research and development. Endovascular treatment continues to evolve with the development of new technologies, providing a less invasive method of blocking or repairing cerebral aneurysms from inside a patient's blood vessels. This approach has shown promising results, with the International Subarachnoid Aneurysm Trial (ISAT) demonstrating superiority for endovascular treatment over neurosurgical methods in some instances [29]. However, the benefits of endovascular intervention over surgery in treating ruptured aneurysms of the anterior circulation remain uncertain, and the choice between endovascular and neurosurgical approaches may depend on the specific characteristics of the aneurysm and the patient [42]. Additionally, a cost-effectiveness analysis found that endovascular treatment may lead to reduced disability rates compared to neurosurgical treatment. Still, the long-term cost-effectiveness and the need for follow-up cerebral angiograms and additional treatments should be considered [43]. Therefore, while endovascular treatment shows promise, selecting the most suitable approach should be based on a thorough evaluation of the individual case, considering the specific characteristics of the aneurysm and the patient's condition.

Blood pressure management challenges and differences: Managing blood pressure in the context of endovascular and neurosurgical approaches for cerebrovascular conditions, such as stroke and aneurysm treatment, presents complex and challenging considerations. In the case of endovascular treatment for acute ischemic stroke, managing blood pressure before, during, and after the procedure is crucial, with specific guidelines and recommendations for different phases of care [34,41]. The absence of specific evidence or guideline recommendations on blood pressure management before, during, and after endovascular thrombectomy for acute ischemic stroke highlights the need for further research and the importance of individualized patient care [41]. Additionally, the benefits of endovascular intervention over surgery in treating ruptured aneurysms of the anterior circulation remain uncertain, emphasizing the need for a thorough evaluation of the available evidence and the specific characteristics of each case [42]. The evolving nature of endovascular treatment and the technological advances in endovascular neurosurgery further underscore the importance of staying abreast of the latest developments in the field to ensure optimal care delivery [44].

Patient-specific factors

Age

Age is pivotal as a patient-specific factor that significantly influences various facets of medical care, encompassing medication dosing, surgical outcomes, and perioperative management. In treating cerebral aneurysms, age becomes a crucial determinant in decisions regarding the selection between endovascular and neurosurgical approaches and the nuanced management of blood pressure during the procedure [45,46]. In the realm of medication dosing, age intricately shapes the pharmacological landscape. Pediatric and geriatric patients manifest distinctive responses compared to adults, necessitating careful consideration in drug dosing. Customizing drug dosages to account for age-specific variations in drug processing and metabolism is imperative. Older individuals, often dealing with renal and hepatic issues, may undergo altered medication processing, highlighting the need for personalized dosing strategies to optimize therapeutic outcomes and minimize potential adverse effects [45].

The impact of age on surgical outcomes is substantial, notably affecting interventions such as coronary artery bypass graft surgery and aneurysm treatment. Older patients may face challenges leading to potentially less favorable outcomes than their younger counterparts. Considering the nuances of age-related physiological changes and comorbidities becomes pivotal in selecting the most appropriate treatment method. This acknowledgment underscores the necessity for personalized approaches, recognizing age as a critical determinant in tailoring interventions to enhance surgical success and overall patient well-being [46]. The influence of age extends into perioperative care, where managing blood pressure becomes a nuanced task, especially in older patients. Sensitivity to medications and an increased susceptibility to complications underscore the importance of age-specific considerations in perioperative blood pressure management. This is particularly evident in endovascular treatments for acute ischemic stroke, where adherence to specific guidelines for blood pressure management before, during, and after the procedure is crucial. Integrating age-related factors into perioperative protocols ensures a more targeted and safer approach, aligning with older individuals' unique needs and responses [39].

Comorbidities

Comorbidities are patient-specific factors that can impact the management and treatment of cerebrovascular conditions, such as stroke and aneurysm treatment. Comorbidity interrelatedness, which refers to how conditions interact in ways that generate clinical complexity, can be a significant challenge in managing patients with multiple chronic conditions [47]. Comorbidities are associated with worse health outcomes, more complex clinical management, and increased healthcare costs [48]. In the context of myelofibrosis, patient-specific comorbidities, such as non-hematopoietic organ dysfunction, have been shown to impact overall survival and should be considered in clinical decision-making [49]. The impact of comorbidities on managing and treating cerebrovascular conditions underscores the importance of individualized patient care and the need to consider patient-specific factors when making treatment decisions.

Aneurysm Characteristics

Patient-specific factors, such as aneurysm characteristics, are crucial in managing cerebral aneurysms. Aneurysm location, size, shape, and hemodynamics are known to be risk factors associated with IA rupture [50,51]. A systematic review and meta-analysis found that aneurysm locations were divided into four categories: anterior cerebral artery, anterior communicating artery, and pericallosal artery [52]. Morphological features, such as size ratio, undulation index, ellipticity index, non-sphericity index, and hemodynamic parameters, such as average wall shear stress (WSS), maximal WSS, low WSS area, average oscillatory shear index (OSI), number of vortices, and relative residence time, have been identified as independent discriminants of aneurysm rupture status [50]. Smoking, hypertension, a history of SAH, sex, and population are also known to be patient-specific clinical factors associated with IA rupture [23,51]. Therefore, a thorough evaluation of the individual case, considering the specific characteristics of the aneurysm and the patient's condition, is essential for selecting the most suitable approach for managing cerebral aneurysms.

Complications and risks

Neurological Complications

Neurological complications can occur as a result of various medical conditions and treatments, including those related to cerebral aneurysms. Complications involving the central and peripheral nervous system are frequently encountered in the intensive care unit, and they can be related to underlying critical illness, pre-existing comorbid conditions, and commonly used life-saving procedures and medications [53]. In the case of cerebral aneurysms, patient-specific factors, such as aneurysm location, size, shape, and hemodynamics, are known to be risk factors associated with IA rupture [54,55]. Additionally, endovascular treatment for cerebral aneurysms carries the risk of bleeding in the brain or loss of blood flow to the brain, which can result in neurological complications [56]. Therefore, a thorough evaluation of the individual case, considering the specific characteristics of the aneurysm and the patient's condition, is essential for selecting the most suitable approach for managing cerebral aneurysms and preventing neurological complications.

Systemic Complications

Hemodynamic instability is a significant concern in endovascular treatments due to their dynamic nature, which introduces the potential for fluctuations in blood pressure and heart rate. These variations pose challenges during the procedure, impacting overall cardiovascular equilibrium. As a result, vigilant monitoring and timely intervention become imperative to maintain stable hemodynamics [57]. Another critical consideration revolves around coagulation and bleeding risks associated with using anticoagulants in endovascular procedures. This elevation in bleeding risk is a pivotal concern during and after interventions. The delicate balance required between preventing thrombosis and avoiding excessive bleeding demands precise management to mitigate potential blood loss and associated complications [57].

The administration of contrast agents, a common practice to enhance imaging in endovascular procedures, introduces the risk of contrast allergy. Allergic reactions, including severe responses like anaphylaxis, can occur. Therefore, a thorough patient assessment, pre-procedural screening, and preparedness to manage potential contrast-related complications are crucial aspects of patient care [57]. Renal impairment is a potential risk associated with the use of contrast agents in endovascular interventions, particularly in individuals with pre-existing kidney disease. Careful consideration and pre-procedural evaluation of renal function are essential to minimize potential adverse effects on renal health [57].

Cardiac complications are also within the spectrum of concerns related to endovascular treatments. These complications can range from cardiac ischemia and arrhythmias to myocardial infarction, emphasizing the intricate interplay between the cardiovascular system and procedural intricacies. Thus, a comprehensive approach to monitoring and managing potential cardiac-related issues during and post-intervention is essential [57]. Although rare, infections can be a post-endovascular procedure complication, especially in cases involving stroke or aneurysm rupture. Maintaining aseptic techniques during the procedure and vigilant post-procedural monitoring for signs of infection are crucial in preventing and addressing potential infectious complications [57]. In the context of aSAH, systemic complications may arise due to sympathetic nervous system activation. These complications include neurogenic pulmonary edema, electrocardiographic changes, troponin elevation, neurogenic stunned myocardium, hyponatremia, and anemia. Recognizing and effectively managing these systemic manifestations are integral to comprehensive care following an aSAH [58].

## Conclusions

In conclusion, this comprehensive review sheds light on the intricate relationship between cerebral perfusion and blood pressure management in neurosurgical and endovascular aneurysm interventions. Key findings underscore the pivotal role of maintaining a delicate balance in blood pressure during these procedures, given the brain's vulnerability to hemodynamic fluctuations. The nuances of cerebral perfusion, governed by autoregulation mechanisms, emphasize the need for a nuanced and personalized approach to hemodynamic control. These insights carry profound implications for clinical practice, emphasizing the importance of adhering to tailored blood pressure targets to minimize ischemic and hemorrhagic risks. In neurosurgery, preoperative and intraoperative strategies must be meticulously calibrated, while endovascular interventions demand heightened awareness during the peri-procedural period. Looking ahead, recommendations for future research underscore the necessity of determining optimal blood pressure targets, advancing monitoring technologies, exploring long-term outcomes, and embracing personalized approaches. As we conclude, this review not only consolidates current knowledge but also serves as a springboard for ongoing research endeavors, aiming to continually refine and elevate the standards of care in the dynamic landscape of neurovascular interventions.
